# Molecular Dynamics Simulations of the Allosteric Modulation of the Adenosine A2a Receptor by a Mini-G Protein

**DOI:** 10.1038/s41598-019-41980-x

**Published:** 2019-04-02

**Authors:** Pedro Renault, Maxime Louet, Jacky Marie, Gilles Labesse, Nicolas Floquet

**Affiliations:** 1 Institut des Biomolécules Max Mousseron (IBMM), CNRS UMR5247, Université de Montpellier, ENSCM, 34090 Montpellier, France; 2 Centre de Biochimie Structurale, Université de Montpellier, CNRS, INSERM, 34090 Montpellier, France

## Abstract

Through their coupling to G proteins, G Protein-Coupled Receptors (GPCRs) trigger cellular responses to various signals. Some recent experiments have interestingly demonstrated that the G protein can also act on the receptor by favoring a closed conformation of its orthosteric site, even in the absence of a bound agonist. In this work, we explored such an allosteric modulation by performing extensive molecular dynamics simulations on the adenosine A2 receptor (A2aR) coupled to the Mini-Gs protein. In the presence of the Mini-Gs, we confirmed a restriction of the receptor’s agonist binding site that can be explained by a modulation of the intrinsic network of contacts of the receptor. Of interest, we observed similar effects with the C-terminal helix of the Mini-Gs, showing that the observed effect on the binding pocket results from direct local contacts with the bound protein partner that cause a rewiring of the whole receptor’s interaction network.

## Introduction

G protein-coupled receptors (GPCRs) constitute the largest family of human membrane receptors and are involved in numerous physiological processes. Among other partners, they can couple to G proteins to trigger signals that result in complex cellular responses^1^. It has been recognized that GPCRs are dynamical molecules that exist as an ensemble of conformations, whose equilibrium can be shifted by their interactions with other molecules^2,3^. Therefore, understanding GPCRs activation and designing drugs to modulate their function require a better comprehension of their dynamical behaviour.

The dynamics of GPCRs and the effect of ligands upon their conformational equilibria have been extensively investigated^4–8^. Computational approaches, including molecular dynamics simulations (conventional as well as enhanced sampling schemes), have contributed to provide a clearer picture of (1) the activation process and (2) the response of GPCRs to different types of ligands (agonists, antagonists or inverse agonists)^9–13^. The simulations have benefited enormously from the structural data accumulated in recent years, that culminated in the structural resolution of complexes formed by the GPCR bound to different heterotrimeric G proteins^14–17^. These structures revealed details of the interaction between receptor and G protein and established a basis for the comprehension of signal transmission from the agonist-bound receptor to the G protein. Accordingly, computational methods have been used to study the allosteric connections between the agonist binding site of the receptor and the nucleotide binding site of the G protein, usually focusing on the modulation of the G protein by the receptor^18,19^. However, in a study recently published by Devree *et al*., experiments with the β2aR have shown that the G protein can also allosterically modulate the receptor even in the absence of agonist^20^. The authors captured the receptor coupled to the G protein but unbound to the agonist and demonstrated that the G protein hinders the association of ligands to the orthosteric site. Moreover, it has also been shown that the G protein slows the dissociation of ligands once they are bound to the site, resulting in the enhanced agonist affinity observed for many GPCRs in the presence of G protein^20^. These observations led to the hypothesis that the G protein stabilizes a closed conformation of the receptor, with a restricted access to the agonist binding site^20^. Based on their experiments and on available crystal structures of receptors coupled to G proteins, the authors of that study proposed a mechanism of signal transmission from the G protein to the orthosteric binding site of the receptor and suggested that this could be a general allosteric mechanism among the class A GPCRs^20^. However, a dynamical view of this modulation is still necessary.

In this work, we proposed to explore the hypothesis of the stabilization of a closed conformation of the receptor by a G protein and to supply a dynamical view of this process.

**Figure 1:**
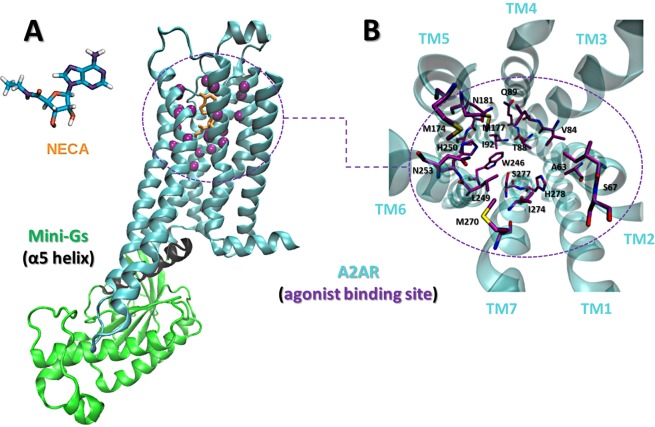
The A2aR:Mini-Gs:NECA complex. (**A**) A2aR (blue) bound to Mini-Gs (green). The Cα’s of residues of the agonist binding site are shown as purple spheres and the C-terminal helix (α5) of Mini-Gs is in black. The agonist NECA is in orange and is also represented separately. (**B**) Detail of the agonist site. Side chains are shown as sticks. The extracellular loops were omitted for clarity.

In particular, we focused on the effect of the G protein upon the unbound receptor and performed molecular dynamics (MD) simulations to study the allosteric signaling from the intracellular partner to the agonist site. We intended to investigate if the G protein was able to stabilize a closed orthosteric site, even in the absence of a bound agonist. Our model system was the adenosine A2 receptor (A2aR), a prototypical class A GPCR, coupled to Mini-Gs (Fig. 1), an engineered protein that binds to A2aR through an interface similar to the one between Gs and β2AR^21,22^.

Although it is reduced to parts of the G protein alpha-subunit, experiments have shown that this mini-protein also causes an increase in agonist affinity, as observed with the full Gs protein^22^. Moreover, the smaller size of Mini-Gs in comparison to the full Gs represented a reduced computational cost and allowed longer simulation times.

The A2aR is a promising therapeutic target as its modulation by selective agonists and antagonists could help in the treatment of many different diseases including neurological disorders (e.g. Parkinson’s disease), cancer and inflammation^23,24^. Also, the structure of A2aR has been obtained in different activation states (inactive^25–30^, active-intermediate^31–33^ and active^22,34^), what makes it useful for the investigation of receptor activation. Therefore, this is a highly relevant model for the study of dynamics and allostery in GPCRs.

## Results

Starting from the crystal structure of the A2aR:Mini-Gs complex in the presence of the agonist NECA (PDB ID: 5G53; Fig. 1), we built four different systems: (1) A2aR; (2) A2aR:Mini-Gs; (3) A2aR bound to Mini-Gs C-terminal α5 helix only (A2aR:α5), since Mini-Gs contacts A2aR mainly through this helix and many experimental studies have also shown that it is able to mimic the full G protein, both functionally and structurally^35,36^; (4) A2aR:Mini-Gs:NECA, that served as a reference for the closed state of the orthosteric site. We emphasize that the initial conformation of the receptor was the same in all systems, as they were built by removing either the ligand, Mini-Gs or both. Two independent simulations of 0.5 μs were performed for each system (a total of 4 μs of simulation) to investigate the effect of the different partners on both the conformation of the agonist binding site and the global contact network of the receptor. The stability of all simulations performed in this study was attested by the backbone RMSD of the receptor, displayed in Fig. S1.

### Mini-Gs or the α5 helix restrict the conformational space of the agonist site

**Figure 2:**
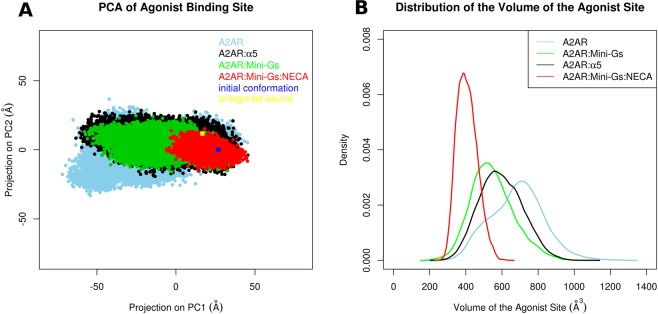
Dynamics and geometry of the agonist site. (**A**) PCA of the cartesian coordinates of the site. The agonist maximally restricted the conformation of the site (red), while the free receptor explored a larger conformational space (light blue); even in the absence of the agonist, Mini-Gs (green) or α5 (black) limited the conformational freedom of the site. The initial conformation of all trajectories (PDB ID 5G53) is represented by a blue square. For reference, an antagonist-bound conformation (PDB ID 5UIG) is also projected (yellow square). (**B**) Probability distribution of the volume of the agonist site. The y-axis indicates the kernel density estimation of the probability density function. The smallest volumes were observed when the agonist was bound (red). The free receptor (blue) explored conformations with the largest volumes. Even in the absence of agonist, Mini-Gs (green) or α5 (black) were able to maintain a reduced volume of the site, in comparison to the free receptor.

We first looked at the conformational freedom of residues of the agonist binding site using a Principal Component Analysis (PCA) of their cartesian coordinates. Residues containing at least one atom within 4.5 Å of the agonist NECA in the crystal structure of the A2aR:Mini-Gs:NECA complex (PDB ID 5G53) were included as part of the binding site. These residues are listed in the Supporting Information (Text S1), their C’αs are shown as purple spheres in Fig. 1A and they are shown in detail in Fig. 1B. Figure 2A shows the projection of the conformations obtained for each studied system in a same space, defined by the first two PCs derived from a concatenated trajectory of the receptor, combining all simulated conditions. The initial conformation of A2aR in all simulations, which corresponds to the receptor bound to the agonist NECA and to Mini-Gs, is also projected onto the same PC subspace and represented in the figure (dark blue square). For reference, the projection of an antagonist-bound conformation is also shown (yellow square). In presence of NECA (red) we observed a maximal restriction of the conformation of the agonist site. This was explained by the rigidity of NECA during the simulations, which limited the flexibility of surrounding residues of the receptor: the average RMSD of NECA relative to the crystal structure was of 1.4 Å. In contrast, in the absence of any partner the A2aR agonist site explored a larger conformational space, visiting not only conformations sampled in the presence of intracellular partners or NECA but also others, apparently inaccessible to the bound forms. The most interesting results came from simulations of the receptor coupled only to the Mini-Gs: a restriction of the agonist site was also observed despite the absence of NECA (green). Even though the site was not as restricted as in the presence of the agonist, it could not reach all the conformations explored by the free receptor. Interestingly, a similar result was obtained when A2aR was bound to the α5 helix (black).

### Mini-Gs or the α5 helix stabilize closed conformations of the agonist site

To investigate if the conformations explored by the distinct systems were associated to different volumes of the agonist site, we measured this volume and calculated its probability distribution in the different conditions. These distributions are illustrated in Fig. 2B. The presence of NECA determined a narrow peak centered at the smallest volume (red). The distribution for the free receptor (blue) was shifted towards higher volumes and was also wider, showing the increased structural variability already indicated by PCA. Coupling to Mini-Gs in the absence of NECA (green) led to an intermediate distribution, where volumes were decreased when compared to the free receptor. Remarkably, a similar result was observed when the α5 helix was bound to A2aR (black). Therefore, Mini-Gs or the α5 helix led not only to a conformational restriction of the agonist site, but also favored more closed conformations of the site in comparison to the uncoupled receptor, even in the absence of agonist.

To test whether the α5 helix could also behave similarly to Mini-Gs in the agonist-bound receptor, we have simulated A2aR bound to NECA and coupled to α5 (A2aR:α5:NECA). In this case, the conformational space of the orthosteric site was as restricted as in the A2aR:Mini-Gs:NECA complex, with volumes comparable to those observed with the full Mini-Gs (Fig. S2). These results suggest that the contacts between A2aR and the α5 helix are an essential ingredient of the allosteric stabilization of the closed forms of the agonist site, with or without the agonist bound to the receptor.

We have also extended the trajectories of A2aR, A2aR:Mini-Gs:NECA and A2aR:Mini-Gs to 2 μs (reaching a total of 12 μs of simulation time for these three systems) and we have found the same pattern of volume distributions (Fig. S3). PCA based on the coordinates of the agonist site performed in these extended trajectories (Fig. S4) also corroborate the results after 500 ns, shown in Fig. 2. Therefore, a 500 ns simulation time was sufficient for observing the effect of Mini-Gs or α5 on the relaxation and geometry of the agonist site.

**Figure 3:**
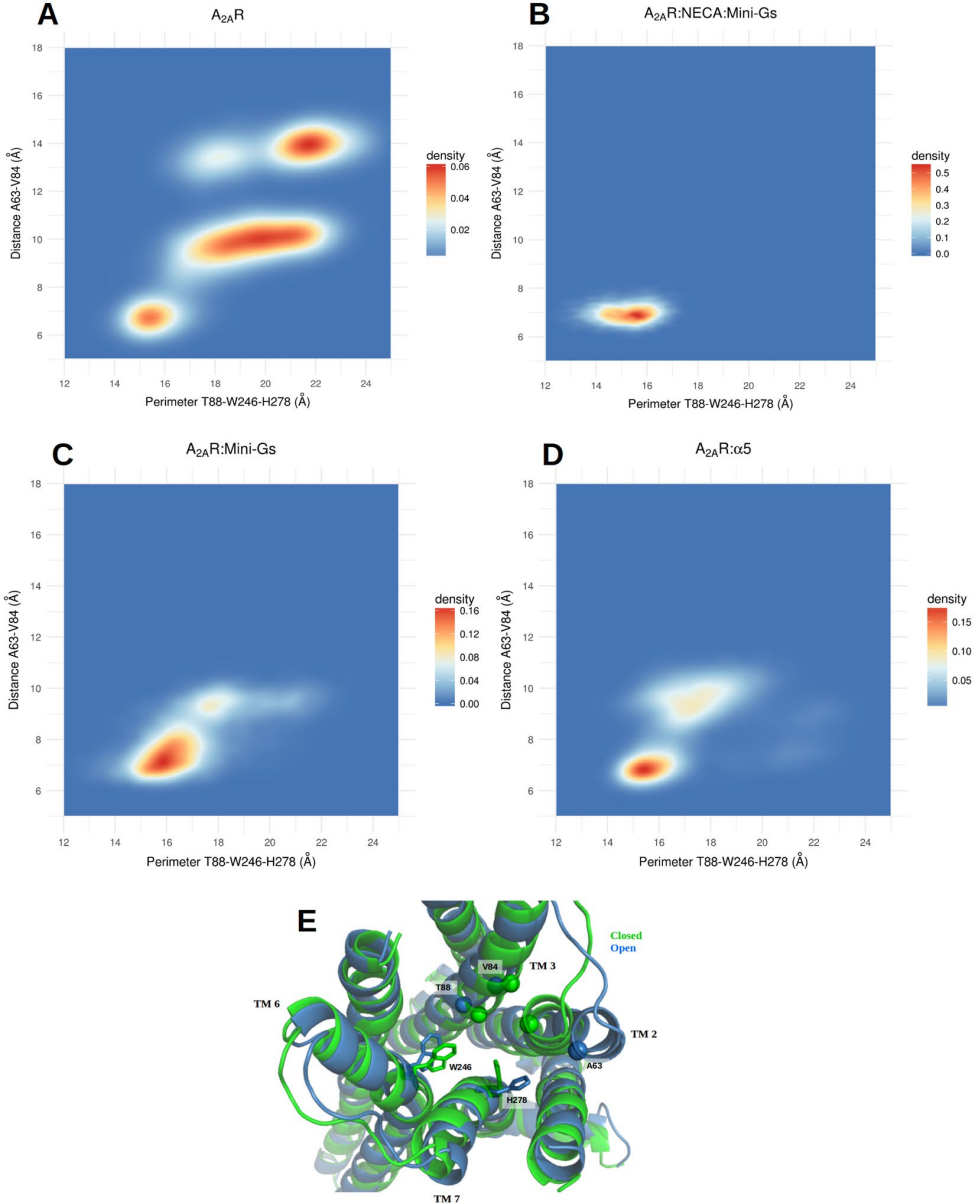
Conformations of the open and closed states of the agonist site. (**A**–**D**) 2D density plots showing the distribution of distances between the Cα’s of ALA 63 (2.61) and VAL 84 (3.32) relative to the perimeter of the triangle formed by the Cα of THR 88 (3.36) and the side chains of TRP 246 (6.48) and HIS 278 (7.43). The free receptor explores closed, intermediate and open states. Mini-Gs or the α5 helix prevent the exploration of the open state and shift the equilibrium towards the agonist-bound conformation; (**E**) Superposition of representative conformations of the closed state of A2aR:Mini-Gs and the open state of A2aR. The side chains of TRP 246 and HIS 278 are shown in licorice representation and the Cα’s of ALA 63 (2.61), VAL 84 (3.32) and THR 88 (3.36) are shown as spheres.

To assess the conformational changes responsible for the differences in the volume, we measured the pairwise distances between the residues of the agonist site in all simulated conditions. We evaluated the distances between the Cα’s and also between the side chains, and determined the Pearson correlation coefficient between every measured distance and the volume. All measured correlations are available as Supplementary Data and their distributions are depicted in histograms in Fig. S5. Backbone and side chain motions played important roles to modify the geometry of the agonist site. Distances between the Cα’s of residues in TM2 and TM3 were the most strongly correlated with the volume, particularly the distance between residues ALA 63 (2.61) and VAL 84 (3.32), with a Pearson correlation coefficient of 0.69 (Fig. S6; the numbers in parenthesis denote the Ballesteros-Weinstein numbering scheme^37^). Higher volumes of the orthosteric site were also associated to a larger distance between the backbones of TM3 and TM7; most notably, the distance between the Cα’s of residues THR 88 (3.36) and HIS 278 (7.43) displayed a correlation coefficient of 0.64 with the volume of the site. HIS 278 was also important because of the conformational changes of its side chain, whose distance to the side chain of TRP 246 (6.48) was significantly associated to variations in the volume of the site (correlation coefficient of 0.63). These distances were indicative of the degree of openness of the site and we studied their distributions in the distinct simulated conditions. The results are illustrated in Fig. 3A–D, with displays of 2D density plots of the distance between the Cα’s of ALA 63 (2.61) and VAL 84 (3.32) relative to the perimeter of a triangle formed by the Cα of THR 88 (3.36) and atoms on the side chains of TRP 246 (6.48) and HIS 278 (7.43) (see the Methods section for details).

In the free receptor (Fig. 3A) it is possible to distinguish a closed state of the site, characterized by a perimeter T88-W246-H278 of approximately 14–16 Å and the distance 63–84 in the range of approximately 6–7 Å; an open state, with the perimeter T88-W246-H278 of ~21–23 Å and the distance A63-V84 of ~13–15 Å; and a set of intermediate conformations, with the triangle formed by T88-W246-H278 assuming a large range of values between ~16 and ~22 Å and the distance A63-V84 of ~10 Å. In contrast, the A2aR:Mini-Gs:NECA system explores only the closed state of the site (Fig. 3B). Interestingly, for A2aR:Mini-Gs and A2aR:α5, the dynamical equilibrium was shifted towards the closed state and although some excursions into intermediate conformations were observed (Fig. 3C,D), the exploration of the open state was abolished in these cases. Therefore, even in the absence of the agonist the intracellular partners contributed to maintain the orthosteric site in the initial, NECA-bound conformation. This indicates a slower relaxation of the agonist site when Mini-Gs or the α5 helix were coupled to the receptor.

Figure 3E shows a superposition of the closed and open states and highlights the structural differences between them. The distance increase between TM2 and TM3 resulted from an outward movement of TM2, that was the single most important contribution to the expansion of the site. Moreover, in the open form of the free receptor TM2 also underwent a rotation (see Fig. S7), that moved ILE 60 (2.58) away from HIS 278 (7.43). By eliminating steric hindrances, the rearrangement of TM2 allowed a relaxation of the side chain of HIS 278 (7.43), that otherwise protruded into the site, contributing to restrict its volume. In agonist-bound forms, HIS 278 contacted NECA and, without the agonist, Mini-Gs or the α5 helix contributed to keep this residue in the NECA-bound conformation by maintaining the closed form of TM2. Our simulations also clearly pointed to the implication of TRP 246 (6.48) in the volume variations of the A2aR agonist site. This is a highly conserved residue known as the “toggle switch” and its role in the activation of GPCRs has been extensively discussed in the literature^38,39^.

Additionally, we also calculated average distance matrices for the entire receptor, based on the distance between Cα’s, in all simulated conditions. To evaluate deviations from the closed conformation of the receptor, we have obtained difference distance matrices, by subtracting the matrix calculated in A2aR:Mini-Gs:NECA from the matrices of every other condition. The results are illustrated in Fig. S8 and clearly showed the contribution of the backbone motion of TM2 to open the receptor. We also paid special attention to a set of distances between 24 pairs of residues that form a consensus scaffold of non-covalent contacts in GPCRs, independent of the activation state^40^. The frequencies of these contacts (distance of less than 4.5 Å between the closest heavy atoms of the residues) in the distinct systems are available in a Supplementary Table. In agreement with the literature^40^, we observed the overall persistence of these contacts throughout the trajectories.

### Mini-Gs or the α5 helix modify the contact network of the receptor

**Figure 4:**
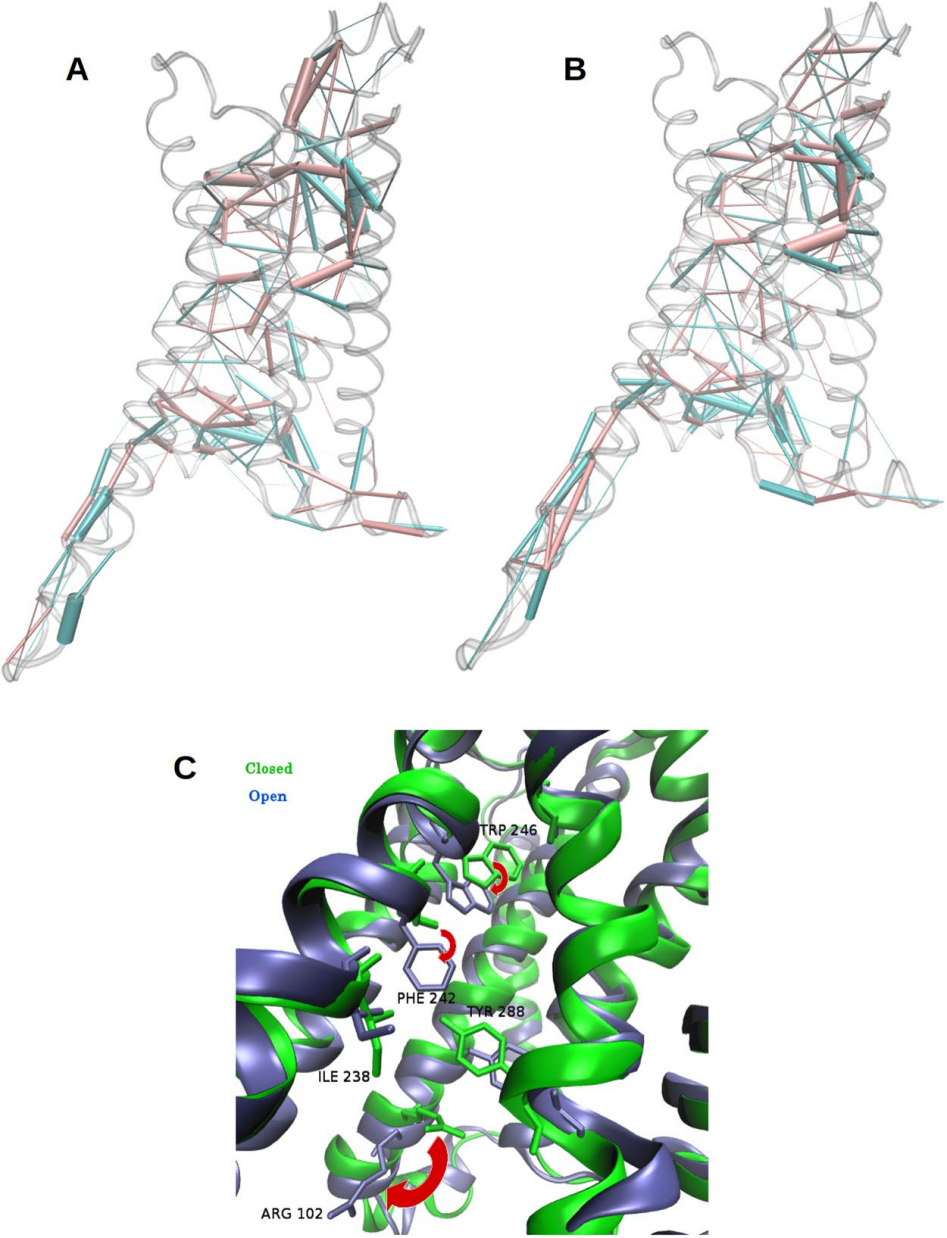
Rearrangement of contacts in A2aR. (**A**) Differences between side chain interactions in A2aR and A2aR:Mini-Gs, revealed by analysis of Protein Structure Networks (PSNs). Pink cylinders: predominant contacts in A2aR:Mini-Gs; blue cylinders: predominant contacts in A2aR. The thickness of the cylinders is proportional to the difference in interaction strength. (**B**) Same analysis for the A2aR:α5 complex. (**C**) Conformational changes of side chains involved in the redistribution of contacts, that extended from the vicinity of the Mini-Gs binding site to the agonist site. Blue: A2aR; green: A2aR:Mini-Gs. The red arrows indicate the relaxation of W246, F242 and R102 upon removal of Mini-Gs.

To investigate more closely how Mini-Gs was allosterically coupled to the othosteric site and led to the reduction of its volume even when the site was empty, we focused on the main structural differences between the closed state of A2aR:Mini-Gs and the open state of the free receptor (see the Methods section for precisions about the selection of these states). For these two groups of conformations, we evaluated the contacts in the entire receptor by means of Protein Structure Networks (PSNs)^41^. This method defines an interaction strength between the side chains of two residues, taking into account the number of atom pairs that are closer than a cutoff distance^28^. Such interaction strengths were computed for all pairs of residues of the receptor, in the closed conformations of A2aR:Mini-Gs and in the open conformations of A2aR. This resulted in two matrices of average interaction strengths, one for each group. The difference between these two matrices highlighted the contribution of Mini-Gs to the structural changes between the two groups, illustrated in Fig. 4A. In this figure, pink cylinders represent interactions that prevail in the closed forms of A2aR:Mini-Gs and blue cylinders indicate predominant interactions in the open A2aR; the diameter of the cylinders is proportional to the difference of interaction strength between the two groups. We draw attention to the main features of this figure: (1) as expected, contacts located in the region of insertion of the α5 helix were strongly modified, but the effect of Mini-Gs extended beyond this region: it caused a global rearrangement of interactions, across the entire receptor; (2) some of the most prominent differences in interaction strength occurred around the agonist site and in the the extracellular loop 2 (ECL 2) (as indicated by the thicker cylinders in these regions). We repeated the same analysis on closed conformations from the simulations of the A2aR:α5 complex with highly similar results (Fig. 4B), indicating that the interaction of the receptor with this helix is a key factor at the origin of the contact redistribution.

Notably, upon removal of Mini-Gs the side chain of ARG 102 (3.50) moved downward, loosing contacts with TYR 288 (7.53), which moved away from ILE 238 (6.40) (Fig. 4C). The latter came into contact with PHE 242 (6.44), which experienced a significant rotameric change and pointed down. This was accompanied by a relaxation of TRP 246 (6.46) (Fig. 4C). Rearrangements of these residues can also be observed in available X-Ray structures, when comparing A2aR coupled to Mini-Gs and uncoupled forms of the receptor. Though our sampling did not capture the complete conversion of the free receptor to its uncoupled conformation, even when the simulations were extended to 2 μs, we observed that ARG 102 (3.50) adopted conformations closer to those found in the inactive A2aR (Fig. S9).

More surprisingly, Mini-Gs or the α5 helix also contributed to strengthen many interactions surrounding the agonist site, with reinforced connections between TM7 and TM2, TM4 and TM5 and TM5 and TM6, 20 to 30 Å from the interaction site of the α5 helix. Residues TRP 246 (6.48) and HIS 278 (7.43), whose importance to the volume of the agonist site was shown (see Fig. 3), were also involved in some of these interactions: in the closed forms, TRP 246 (6.48) maintained contact with SER 277 (7.42) and HIS 278 (7.43) interacted more closely with ILE 60 (2.58), thus contributing to tighten the connections between TM6 and TM7 and between TM7 and TM2. We also observed that coupling to either partner was associated to a modification in the pattern of interactions of the ECL 2, with stronger contacts rigidifying part of this loop (Fig. S10).

**Figure 5:**
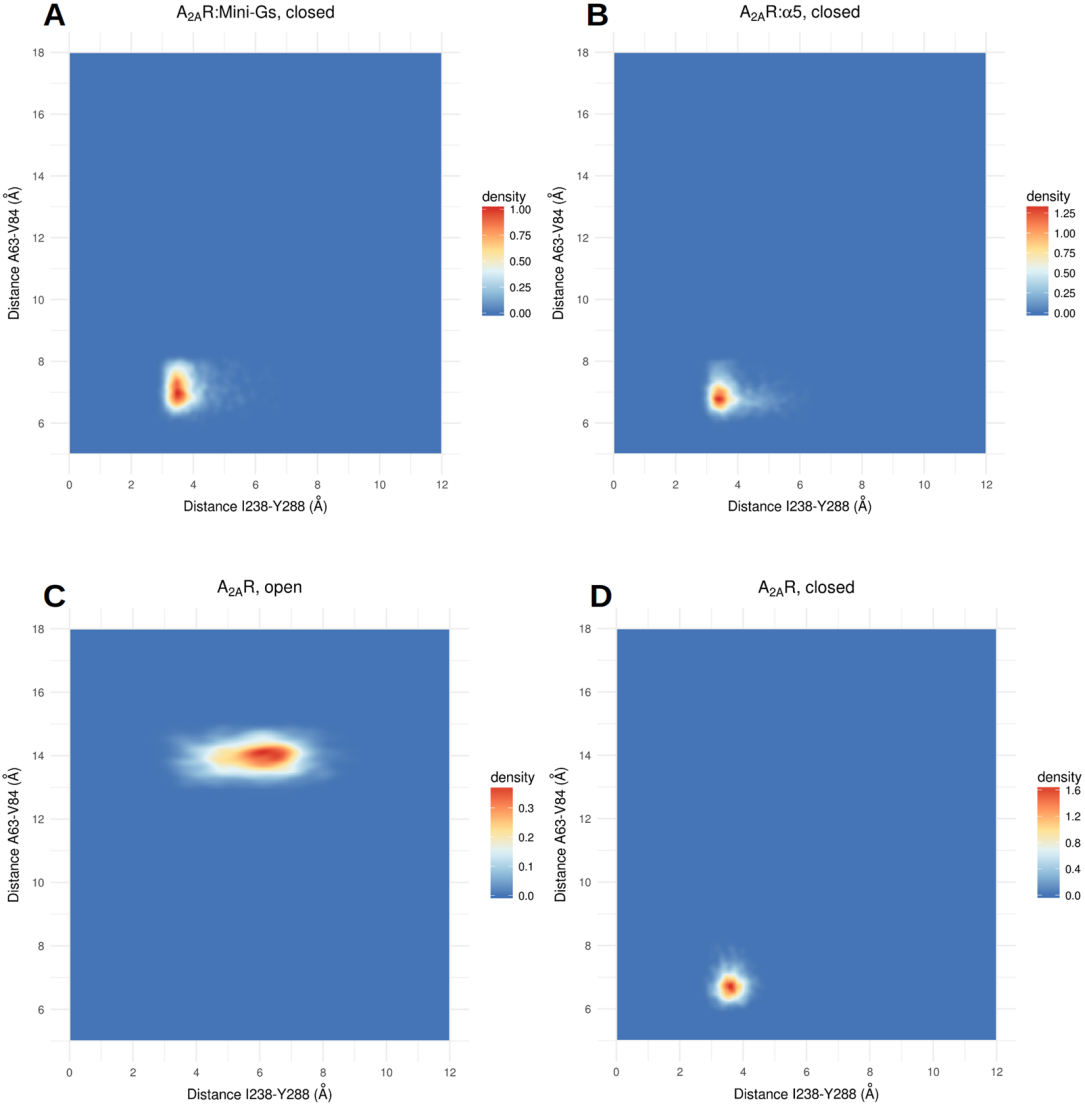
Coupling between the Mini-Gs and agonist binding sites. (**A**–**D**) 2D density plots of the distances between the Cα’s of residues A63 (2.61) and V84 (3.32), in the agonist site, and between the side chains of I238 (6.40) and Y288 (7.53), close to the region of insertion of the α5 helix. The closed state of the site is associated to a shorter I238-Y288 distance, even in the free receptor.

As another evidence for the global and long-range effects of Mini-Gs or α5 helix binding, Fig. 5 shows 2D density plots that reveal the correlation between the conformation of residues next to the Mini-Gs binding site and the distance between TM2 and TM3, that in turn was determinant for the volume of the agonist site. The contacts between the side chains of TYR 288 (7.53) and ILE 238 (6.40) were enhanced in A2aR:Mini-Gs or A2aR:α5 relative to the free receptor and the closer interaction of these residues was associated to shorter distances between the Cα’s of ALA 63 (2.61) and VAL 84 (3.32) in the closed forms of the complexes (Fig. 5A,B). In contrast, in the open state of A2aR a larger 238–288 distance was accompanied by a greater separation between TM2 and TM3 (that resulted in higher volumes of the agonist site; Fig. 5C). Interestingly, when the free receptor explored the closed state, the distance between TYR 288 (7.53) and ILE 238 (6.40) was also reduced (Fig. 5D). This result suggests that Mini-Gs or the α5 helix stabilize conformations of residues close to the intracellular domain that allosterically favor the closed conformation of the agonist site.

To further explore this hypothesis we simulated a double mutant of A2aR, I238A/Y288A, coupled to the α5 helix and measured the volume distribution of the agonist site. In this case, α5 lost the ability to stabilize the closed conformation of the site. The mutated receptor visited open and intermediate states, with large values of the T88-W246-H278 perimeter, while the closed conformation was suppressed. In comparison to A2aR:α5, a pronounced outward motion of TM2 also contributed to open the receptor. These results are shown in Figs S11 and S12 and reinforced the suggestion that the local rearrangement of contacts in the region of interaction with α5 triggers global changes that reach the orthosteric site.

**Figure 6:**
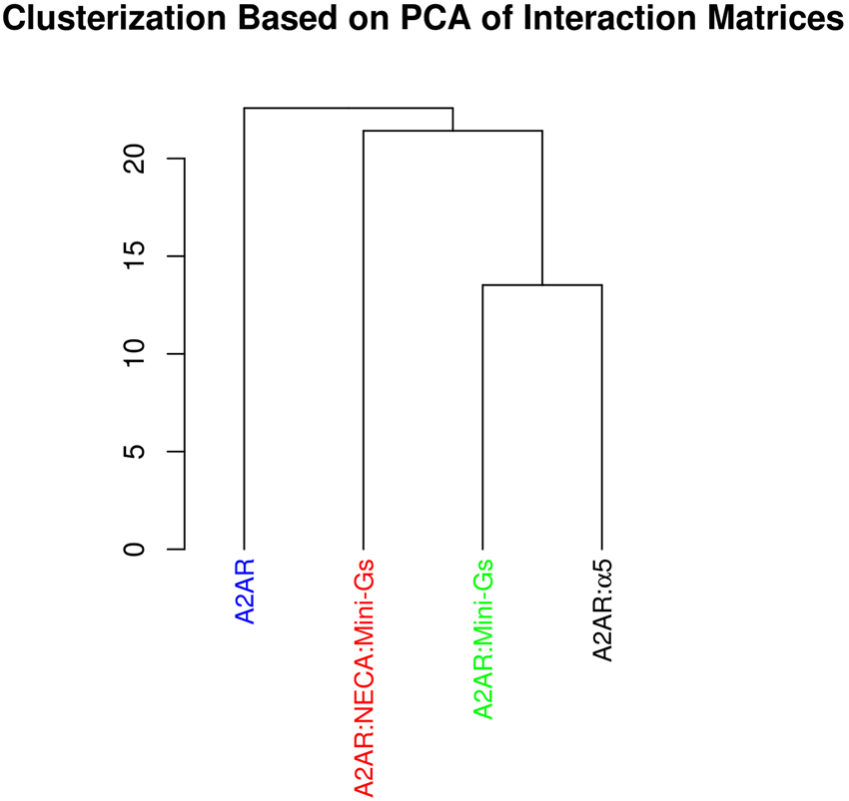
Principal Component Analysis of matrices of average interaction strength of A2aR. Trajectories of A2aR coupled to Mini-Gs or to the α5 helix in the absence of NECA were grouped together, both being closer to the NECA bound form than to the free receptor. This indicates that α5 could maintain the pattern of contacts of the Mini-Gs coupled receptor.

Finally, we also built other PSNs of the receptor, taking into account the whole trajectories and not only conformations from specific states. PSNs were then computed for each of the four different studied systems. After being subjected to PCA, the matrices of average interaction strengths were clustered, confirming that the α5 helix was able to mimic the global effect of the Mini-Gs with respect to the pattern of contacts (Fig. 6).

## Discussion

In this work we employed molecular dynamics simulations to study the allosteric effects of the Mini-Gs protein or its C-terminal α5 helix upon the A2 adenosine receptor. Our simulations confirmed the hypothesis that the G-protein stabilizes a closed form of the receptor even in the absence of agonist and showed that Mini-Gs or α5 were able to shift the dynamical equilibrium towards a closed conformation of the orthosteric site, as suggested by previous experiments^20^.

Considering that all our simulations started from the closed state of the receptor, Mini-Gs or α5 prevented or slowed down a complete relaxation of the agonist site towards an open conformation, even in the absence of ligand. The fact that α5 was able to reproduce the global effect of Mini-Gs suggests that the reorganization of contacts in the G protein binding site is essential to trigger the allosteric signal. It is also consistent with the experimental observation that an antibody that mimics the G protein also leads to a closed conformation of the agonist site^20^. The G protein, the α5 helix and the antibody share the same binding site of the receptor, possibly leading to similar local rearrangements of contacts that eventually affect the entire receptor.

In our simulations, this local rearrangement involved stronger interactions between I238 (6.40) and Y288 (7.53) in the presence of Mini-Gs or α5. Simulations with the double mutant I238A/Y288A confirmed the importance of this contact to the allosteric effect, since its disruption in A2aR:α5 abolished the closed state of the receptor. Y288 is part of the conserved NpxxY motif and, together with Y197, is possibly involved in an activation switch in class A GPCRs^42^. In the A3R, the Y288F mutation led to a 30-fold reduction in potency of a highly selective agonist, suggesting a role of this residue in the dynamical equilibrium of the receptor^43^. The mutation I238A in A2aR led to an increased thermostability of agonist- and inverse-agonist-bound conformations^44^, indicating that this residue can also be important for conformational equilibrium. Experiments with the double mutant I238A/Y288A would be valuable to confirm the pertinence of this theoretical model.

Our analyses showed that an important effect of Mini-Gs was the strenghtening of contacts in ECL2, reducing its flexibility. The roles of ECL2 in ligand selectivity and stabilization of agonist-bound conformations in A2aR have been previously discussed^45^. Our findings suggest that the allosteric effect of the G-protein on ECL2 could contribute to such stabilisation. In particular, E169 is known to be involved in ligand binding^42^ and its interaction with the agonist could be favored by a reduced flexibility of ECL2.

Although it is tempting to discuss our observations in the framework of allosteric communication pathways, we avoided this description in view of results that point to the technical and conceptual difficulties in defining these paths. Hilser and co-workers have argued about the limitations of the concept of pathways in the context of an ensemble view of allostery^46,47^. More recently, simulations permitted a time-resolved study of communication in an allosteric protein, revealing that different trajectories had significant heterogeneity in the propagation of the allosteric signal and that nonlocal and simultaneous structural changes could occur in a single trajectory^48^. Our sampling was not extensive enough for such a detailed study of the precise temporal order of conformational changes. This limited sampling was also reflected in the incomplete relaxation of the free receptor to its inactive conformation, even when the simulations were extended to 2 μs. Nevertheless, we captured rearrangements of residues such as W246, F242 and R102, this last one adopting conformations closer to those found in the inactive A2aR.

Moreover, our results suggest that the observed allosteric effect could result from a complex interplay between the entire network of contacts, that is globally rewired by Mini-Gs or the α5 helix. In spite of this complexity, it was possible to identify interactions that were stabilized in the different tested conditions, even if distinct allosteric pathways or mechanisms could contribute to such stabilization.

Our observations suggest that the closed state would be further stabilized by the binding of the agonist. Therefore, similarly to an agonist that shifts the equilibrium towards the active conformation, that is stabilized by the G protein, the G protein could select conformations more prone to interact favorably with the agonist, that would stabilize them when binding to the orthosteric site. This perspective could be particularly relevant in the study of receptor-ligand interactions in cases where the GPCR:G protein complex is pre-assembled, as recently shown for the ghrelin receptor^49^. In these situations, docking experiments, for example, could benefit from an appropriate selection of more closed conformations of the receptor.

It has been suggested that a common allosteric mechanism could operate on class A GPCRs in order to close the agonist site upon coupling to the G protein^20^. This hypothesis cannot be excluded by our results, but they also point to the importance of considering the specificities of the distinct receptors. For example, in recent simulations, Miao and McCammon verified that the reduction in the volume of the orthosteric site of the M2 muscarinic receptor was associated to backbone motions of TM3, TM6 and TM7^50^. In our simulations of the A2aR, the volume was mainly affected by backbone motions of TM2 and, to a lesser extent, TM3 and TM7, with an important contribution of side chains, too. Even if the underlying mechanisms may differ, the theoretical and experimental results available so far point to the role of G protein in restricting the size of the orthosteric site of the receptor.

While this paper was under review, Lee *et al*.^51^ published an article about the stabilisation of the active state of A2aR by the G protein. They performed molecular dynamics simulations on four agonist-bound conformations of the receptor, including the complex with Mini-Gs. In contrast to our own study, they have not studied ligand-free forms of the receptor and carefullly evaluated the interaction between the protein and the agonist in each case. They observed that coupling of Mini-Gs was associated to tighter contacts with the agonist and more favorable binding free energies^51^. In agreement with our findings, they point to the stabilisation of a closed conformation of orthosteric site by Mini-Gs^51^. They have also reported a stronger allosteric coupling between extra- and intracellular regions in the presence of Mini-Gs, mediated through a high level of correlated motions mainly in TM5^51^. In our investigation, we pointed to important side chain rearrangemets in TM6 (Fig. 4C); however, we note that we analysed a distinct situation: instead of correlated motions in a system, our residue interaction analysis looked for differences in the conformations of side chains between A2aR and A2aR:Mini-Gs (or A2aR:α5). Despite the different approaches, both of our studies demonstrated an allosteric effect exerted by the G protein and the importance of dynamics in its evaluation.

## Methods

### Model preparation

Models were based on the cystal structure of A2aR bound to Mini-Gs, PDB id: 5G53, pre-aligned in the Orientations of Proteins in Membranes database^52^. The chain B of this file was the basis for the A2aR construction and chain D was chosen to build the Mini-Gs model. Addition of missing residues and loop refinement were performed with Modeller^53^. Missing residues in the receptor were located in the intracellular loop 3 (ICL3) region (residues 208–233); in Mini-Gs, residues 224–239 and a stretch of 15 aminoacids between residues 61 and 208 were also missing (note that the numbers refer to the numbering scheme in the original pdb file, 5G53).

Models for A2aR and Mini-Gs were built separately and reassembled through structural alignment to the original pdb file (in Pymol). The double mutant of the receptor (I238A/Y288A) was prepared using the Pymol Mutagenesis Wizard tool. GDP, initially bound to Mini-Gs, and NECA were included in the final model of the A2aR:Mini-Gs:NECA complex. This model was the starting point of the other simulated systems, constructed by removal of the corresponding components.

Models were inserted in 100% POPC membranes, solvated and ionized up to a concentrarion of 0.15 M NaCl using the Charmm-gui web server^54^. Systems containing Mini-Gs were composed of ~105000 atoms (~70000 water atoms and ~27000 lipid atoms in ~200 POPC molecules); systems without Mini-Gs or with the receptor bound to the α5 helix were composed of ~79000 atoms (~50000 water atoms and ~23500 lipid atoms in ~175 POPC molecules).

### Molecular Dynamics Simulations

Two completely independent replicas were launched for each system. Systems were equilibrated following the previously described CHARMM-GUI protocol^55,56^. Briefly, after energy minimization, successive steps of NVT and NPT (300 K, 1 bar) MD were performed, with progressive removal of position restraints applied to the membrane and protein atoms. Then, simulations were run for 600 ns (NPT ensemble, 300 K, 1 bar) and the first 100 ns were considered as equilibration and discarded; the last 500 ns of each replica were retained for analysis. Furthermore, for all systems except A2aR:α5 and the double mutant of A2aR coupled to α5, production simulations were extended until 2.0 μs.

Proteins, lipids and ions were described by the CHARMM36 force field^57–59^ and the parameters for GDP and NECA were obtained with the CGenFF force field^60^, through the CHARMM-GUI interface. A time step of 2 fs was used in the production phase and PME (Particle Mesh Ewald)^61^ was employed for the treatment of long-range electrostatic interactions, with the application of a switch function between 1.0 and 1.2 nm. Temperature was kept at 300 K by the Nose-Hoover scheme^62,63^, using a time constant for coupling of 1 ps. Pressure was maintained at 1 bar by a semi-isotropic Parrinello-Rahman barostat^64^, with coupling time constant of 5 ps and compressibility of 4.5 × 10^−5^ bar^−1^. All bonds to hydrogen atoms were constrained by the LINCS algorithm^65^. All simulations were performed with the Gromacs 5.1.4 software^66^.

### Preparation for Analysis

The coordinates of the receptor were extracted from all trajectories and concatenated. All conformations of the receptor in this “super trajectory”, containing snapshots simulated in all conditions, were superimposed according to a procedure that identifies a rigid core formed by the most invariant regions in terms of dynamics^67^. Only the backbone of the residues was considered in this alignment procedure. The residues of the receptor identified as belonging to this most invariant core are listed in the Text S2. This core was used as the reference for the alignment of all snapshots and the initial model, based on the crystal structure (PDB id: 5G53).

### Principal Component Analysis of the Residues of the Agonist Binding Site

The concatenated and aligned trajectory of the receptor was submitted to a Principal Component Analysis, consisting in the diagonalization of the covariance matrix, whose elements are:

$${{\rm{C}}}_{{\rm{ij}}}=\langle ({{\bf{r}}}_{{\rm{i}}}-\langle {{\bf{r}}}_{{\rm{i}}}\rangle ).({{\bf{r}}}_{{\rm{j}}}-\langle {{\bf{r}}}_{{\rm{j}}}\rangle )\rangle $$

where i and j denote all pairs of the 3N Cartesian coordinates of the N atoms of the residues of the agonist site, including their side chains. The vector **r**_i_ indicates the instantaneous value of coordinate i and 〈**r**_i_〉 is the average value of this coordinate in the ensemble of conformations.

The coordinates of the selected residues in the snapshots corresponding to each individual trajectory were then projected on the space of the respective first two principal components. These analyses were performed with the Gromacs software^66^.

### Distances between residues

The Mdtraj software^68^ was used to measure the Euclidean distance between atoms. For a given pair of residues, two distances were measured in every trajectory frame: (i) between the Cα’s and (ii) between the side chains. In the latter case, it consisted in the distance between the closest heavy atoms in the side chains. To establish the perimeter of the triangle formed by residues 88, 246 and 278, we have additionally determined the distances between the CH2 atom in W246 and the CD2 atom in H278 (the closest side chain atoms in the initial conformation, extracted from pdb file 5G53) and also between the Cα of T88 and each one of these atoms.

### Definition of open and closed states of the receptor

The definition of states was based on the distance between the Cα’s of residues ALA 63 (2.61) and VAL 84 (3.32) and the perimeter of the triangle formed by the Cα of THR 88 (3.36), the CH2 atom of TRP 246 (6.48) and the CD2 atom in HIS 278 (7.43). The following criteria were used to define the states:

Closed: perimeter T88-W246-H278 < 16 Å and distance A63-V84 < 8 Å

Open: perimeter T88-W246-H278 > 21 Å and distance A63-V84 > 13 Å

Otherwise, the states were considered as intermediate.

### Volume of the Agonist Site

Direct estimations of the volume of the agonist site were obtained with the Epock software^69^. Before volume calculation, this program requires the definition of a region of interest (the “maximum encompassing region”). This region was defined as a sphere of 10 Å radius, centered in the geometric center of the residues of the agonist site, listed in the Supporting Information (Text S1). Please refer to the original publication for further details about the method^69^.

### Protein Structure Networks

PSNs were obtained by the method introduced by Brinda and Vishveshwara^41^, using the Wordom software^70^. In this approach, an interaction strength between two amino acid side chains i and j is given by:

$${{\rm{I}}}_{{\rm{ij}}}={({\rm{n}}}_{{\rm{ij}}}/\mathrm{sqrt}\,{({\rm{N}}}_{{\rm{i}}}\times {{\rm{N}}}_{{\rm{j}}}))\times \mathrm{100},$$

where the result is expressed as a percentage. n_ij_ is the number of atom pairs between the side chains that are closer than a cutoff, that was chosen as 4.5 Å; N_i_ and N_j_ are normalization factors that depend on the types of residues i and j (details in the original publication). Residues separated by 3 or less residues in the sequence were considered adjacent and skipped in the contact calculation. PSNs were calculated for all conformations in a given condition, resulting in average interaction strengths over the trajectories. Then, for each condition, one matrix with the interaction strengths between all residue pairs was obtained. These were the matrices used in subsequent analyses.

### PCA and clusterization of interaction strenght matrices

Interaction strenght matrices calculated for the four simulated conditions (A2aR, A2aR:NECA, A2aR:Mini-Gs, A2aR:α5) were subjected to Principal Component Analysis with the Bio3d software^67^.The matrices were then clustered in PC space with the single linkage method.

## Supplementary information

Supplementary information

Dataset 1

Dataset 2

Data set 3
