# Supplementary material for: Molecular Dynamics Simulations of the Allosteric Modulation of the Adenosine A2a Receptor by a Mini-G Protein
Source: Sci Rep. 2019 Apr 2;9:5495. doi: 10.1038/s41598-019-41980-x (PMC6445292; doi:10.1038/s41598-019-41980-x)
Supplement: Supplementary file 1 — Supplementary information [file 41598_2019_41980_MOESM1_ESM.pdf]

Supporting information for:

Molecular Dynamics Simulations of the Allosteric Modulation of the Adenosine A2A Receptor by a Mini-G Protein

Pedro Renault[1,2], Maxime Louet[1], Jacky Marie[1], Gilles Labesse[2] and Nicolas Floquet[1]

1 Institut des Biomolécules Max Mousseron (IBMM), CNRS UMR5247, Université de Montpellier, ENSCM, 34090 Montpellier, France.

2 Centre de Biochimie Structurale, Université de Montpellier, CNRS, INSERM, 34090 Montpellier, France.

This section includes:
Texts S1-S2 and Figures S1-S6.

SUPPORTING INFORMATION TEXT

Text S1. **Residues of the agonist binding site**. Residues containing at least one atom within 4.5 Å of the agonist NECA in the crystal structure of the A2AR:Mini-Gs:NECA complex (PDB ID 5G53) were included as part of the binding site. These residues are ALA 63, SER 67, VAL 84, LEU 85, THR 88, GLN 89, ILE 92, PHE 168, GLU 169, MET 174, MET 177, ASN 181, CYS 185, VAL 186, TRP 246, LEU 249, HIS 250, ASN 253, MET 270, ILE 274, SER 277 and HIS 278.

Text S2. **Residues considered for the alignment of the trajectories**. The residues of the most invariant core were: 18-33; 40-53; 94-107; 118-126; 192-210; 219-241; 281-291 (the numbers refer to the pdb 5G53).

SUPPORTING INFORMATION FIGURES

Figure S1. RMSD of A2AR in the different systems indicate the stability of the simulations. A) A2AR, B) A2AR:Mini-Gs:NECA, C) A2AR:Mini-Gs, D) A2AR:α5, E) A2AR:α5:NECA, F) A2AR_I238A/Y288A:α5. Calculations were based on receptor backbone atoms, considering the conformation of the X-ray structure (PDB ID 5G53) as a reference. The results for each replica are shown separately. Higher values and fluctuations were observed for the free A2AR, consistent with the increased mobility of the receptor in this case.

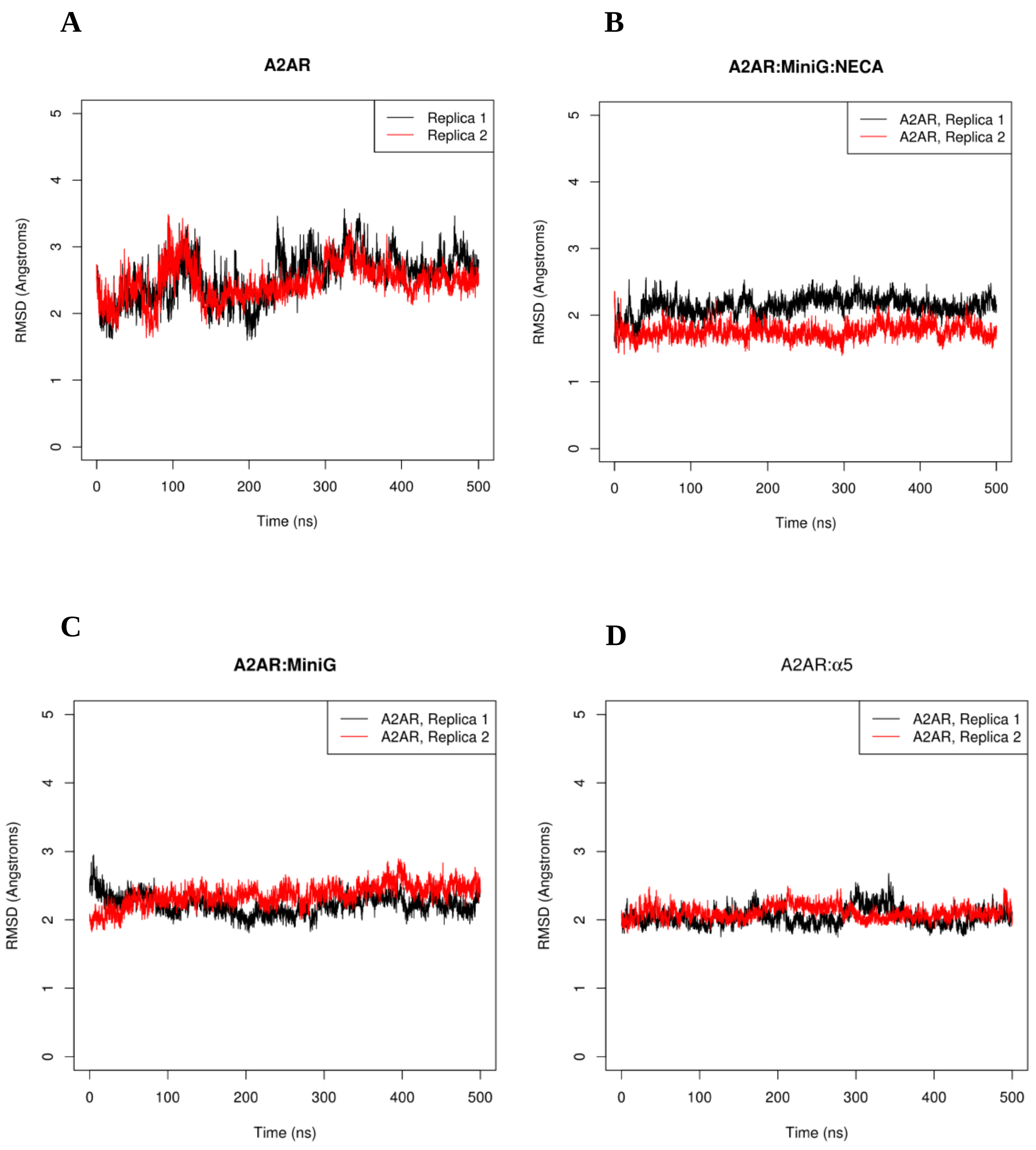

E

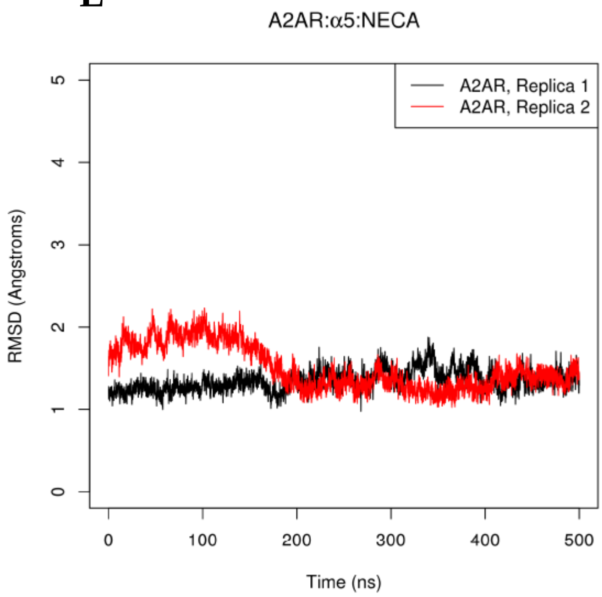
A2AR:α5:NECA
A2AR, Replica 1
A2AR, Replica 2
RMSD (Angstroms)
Time (ns)
0
1
2
3
4
5
0
100
200
300
400
500

F

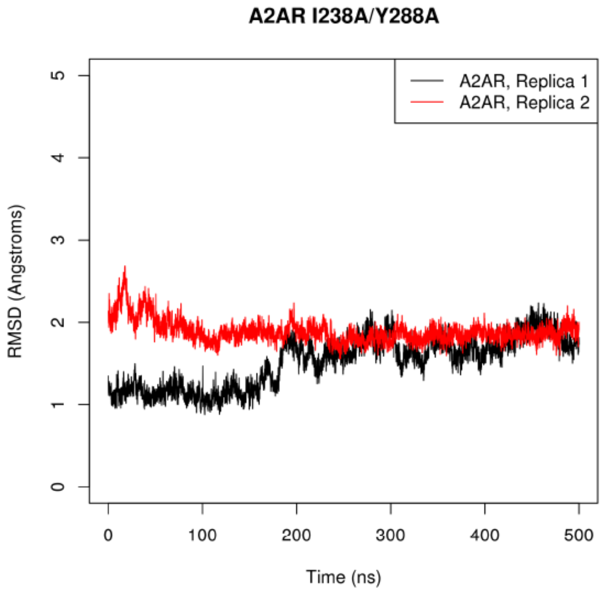
A2AR I238A/Y288A
A2AR, Replica 1
A2AR, Replica 2
RMSD (Angstroms)
Time (ns)
0
1
2
3
4
5
0
100
200
300
400
500

Figure S2. PCA and distribution of volume of the agonist binding site in distinct simulation conditions, including the A2AR:α5:NECA system (orange). The other curves are those shown in figure 2B of the main text. The α5 helix has an effect similar to the full Mini-Gs in restricting the conformational space and stabilizing a reduced volume of the orthosteric site.

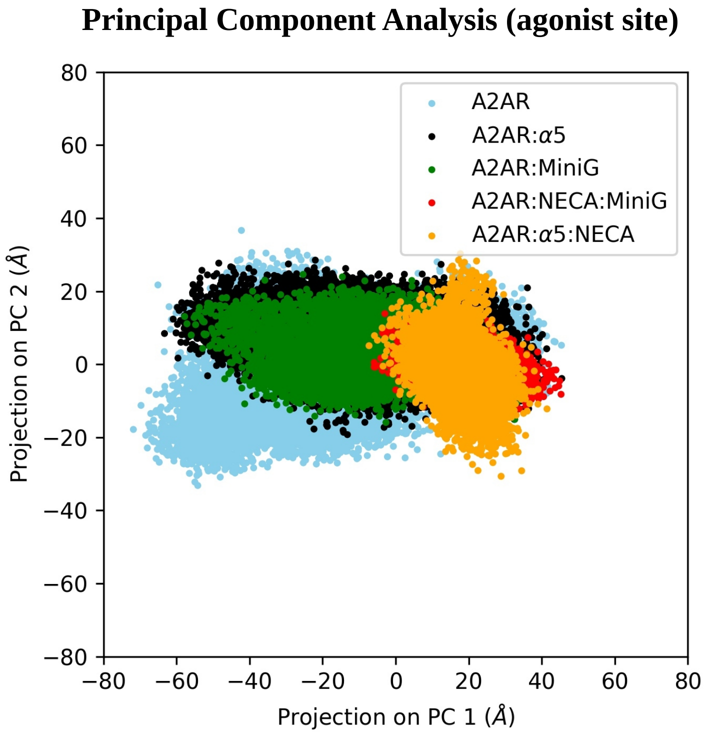

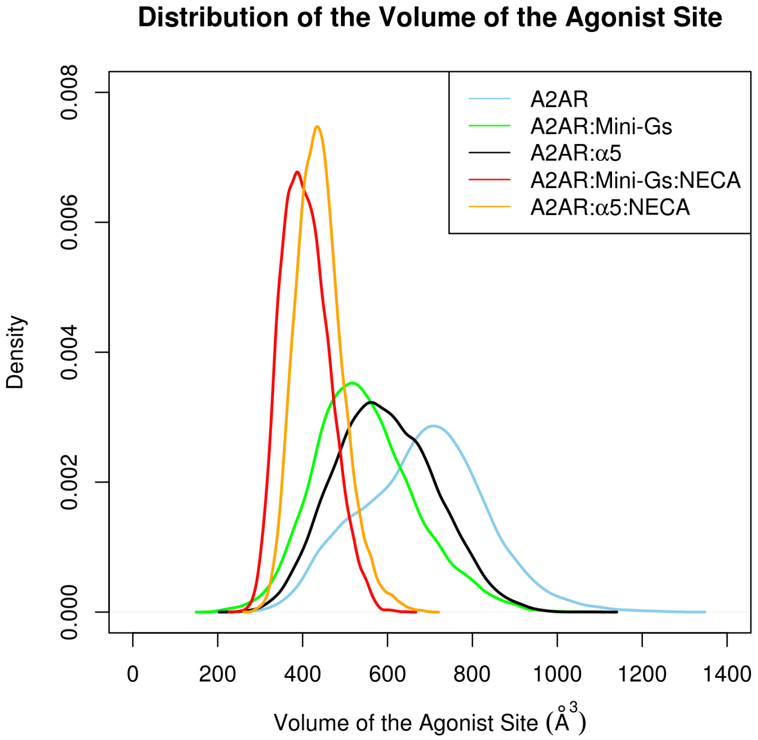

Figure S3. Distribution of the volume of the agonist binding site in distinct simulated conditions, with the exception of A2AR:α5. For a given condition, the distribution was computed from two independent trajectories of 2.0 μs each. The smallest volumes were observed when the agonist was bound (red). The free receptor (blue) explored conformations with the largest volumes. Even in the absence of agonist, Mini-Gs (green) or α5 (black) shifted the equilibrium towards reduced volumes of the site, in comparison to the free receptor. We observed the same pattern of distributions as in figure 2B of the main text, that shows results obtained after 500 ns.

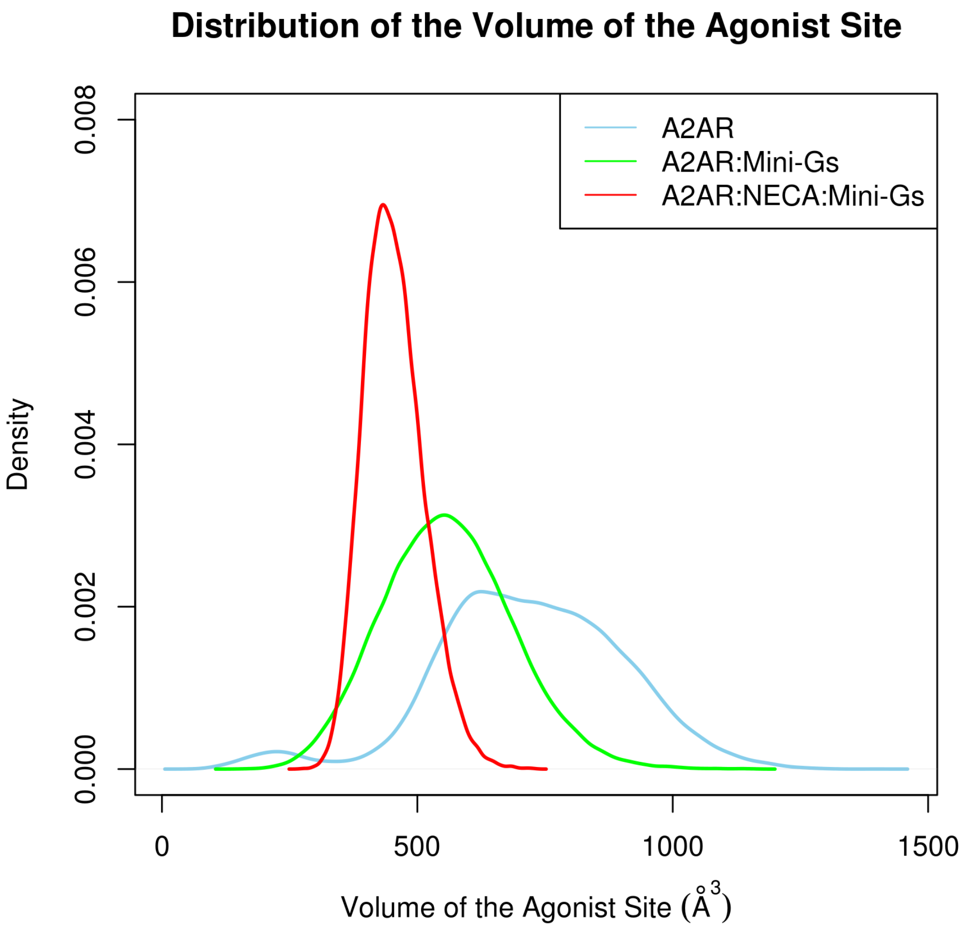

Figure S4. PCA of the cartesian coordinates of the agonist binding site, computed from two independent trajectories of 2.0 μs for each condition. Maximal conformational restriction of the agonist site was observed in the presence of NECA (red). The free A2AR explored a larger space (blue), while Mini-Gs limited the conformational freedom of the site, even in the absence of agonist (green). These results confirm the observations made after 500 ns of simulation for each trajectory, shown in figure 2A of the main text.

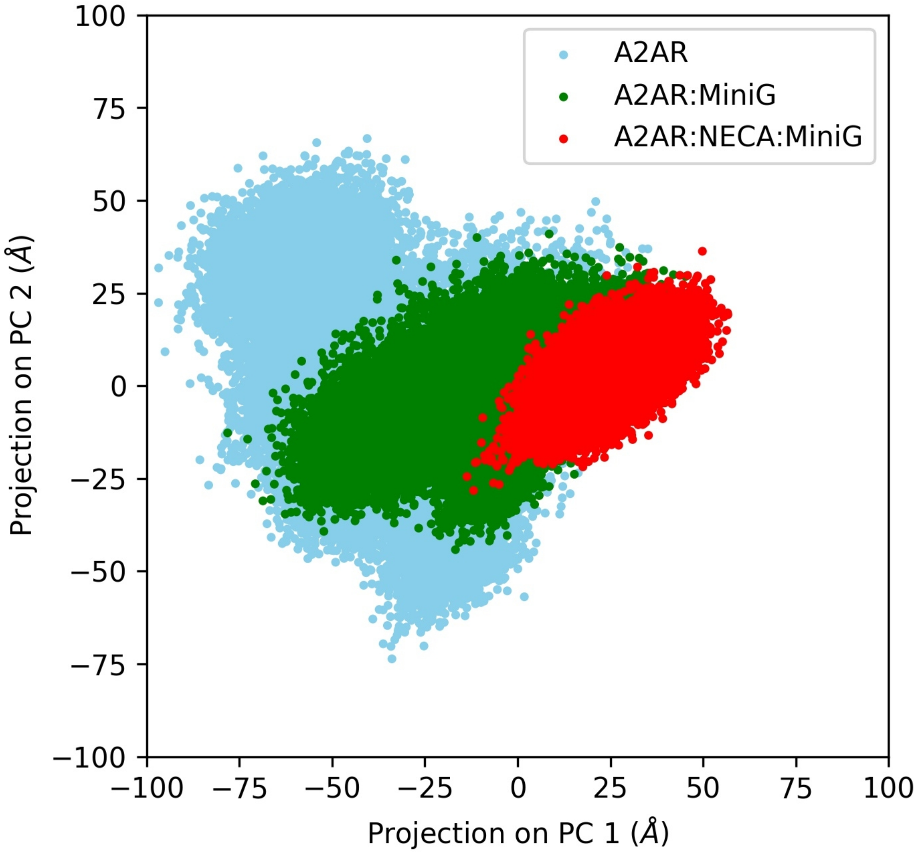

Figure S5. Distributions of correlations between pairwise distances in the agonist site and the volume of the site. A) Distances between Cα's. B) Distances bewteen side chains. Note that most of the correlations are in the range 0.4-0.5 and many pairwise distances are not correlated to the volume. We have studied in detail distances with the highest correlation with the volume in each case (between Cα's of residues A63-V84 and between the side chains of W246-H278).

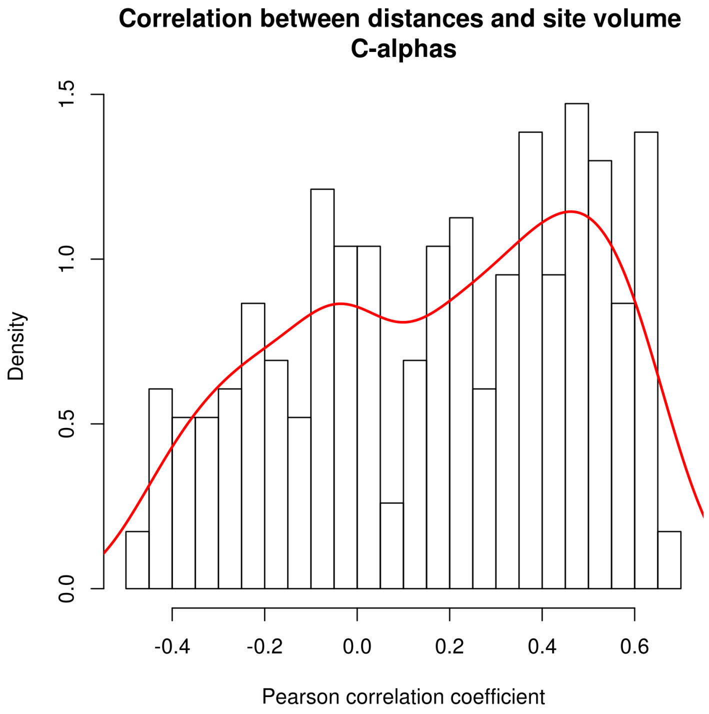

A

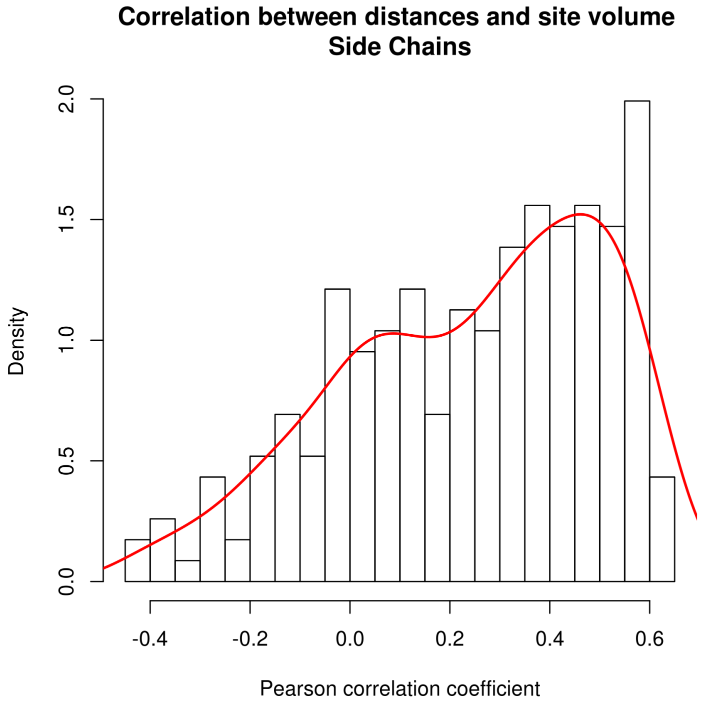

B

Figure S6. Correlation between the Cα A63-V84 distance and the volume of the agonist site. We measured a Pearson correlation coefficient of 0.69. A line of best fit is depicted in red.

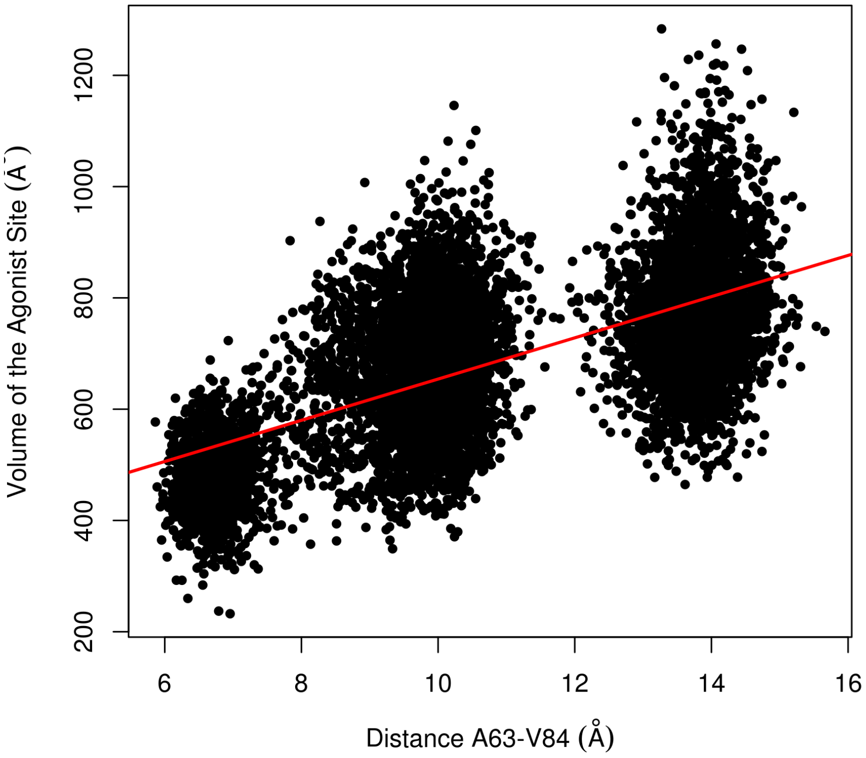

Figure S7. Rotation of TM2 in the closed and open conformations of the receptor, relative to its orientation in the crystal structure of the $A_{2A}R$:NECA:Mini-Gs complex. In the closed state of $A_{2A}R$:Mini-Gs and $A_{2A}R$:α5, TM2 remained in its agonist bound orientation (average rotation angle ~ 0°). In the open state, explored by the free $A_{2A}R$, in addition to the outward movement, TM2 underwent an average rotation of approximately 20°. The dark blue lines in the center and outside of the circle indicate the initial and final values of the rotation angle, respectively. The red line indicates the average value. The calculation was performed with the software Trajelix[1], as implemented in the Simulaid program[2].

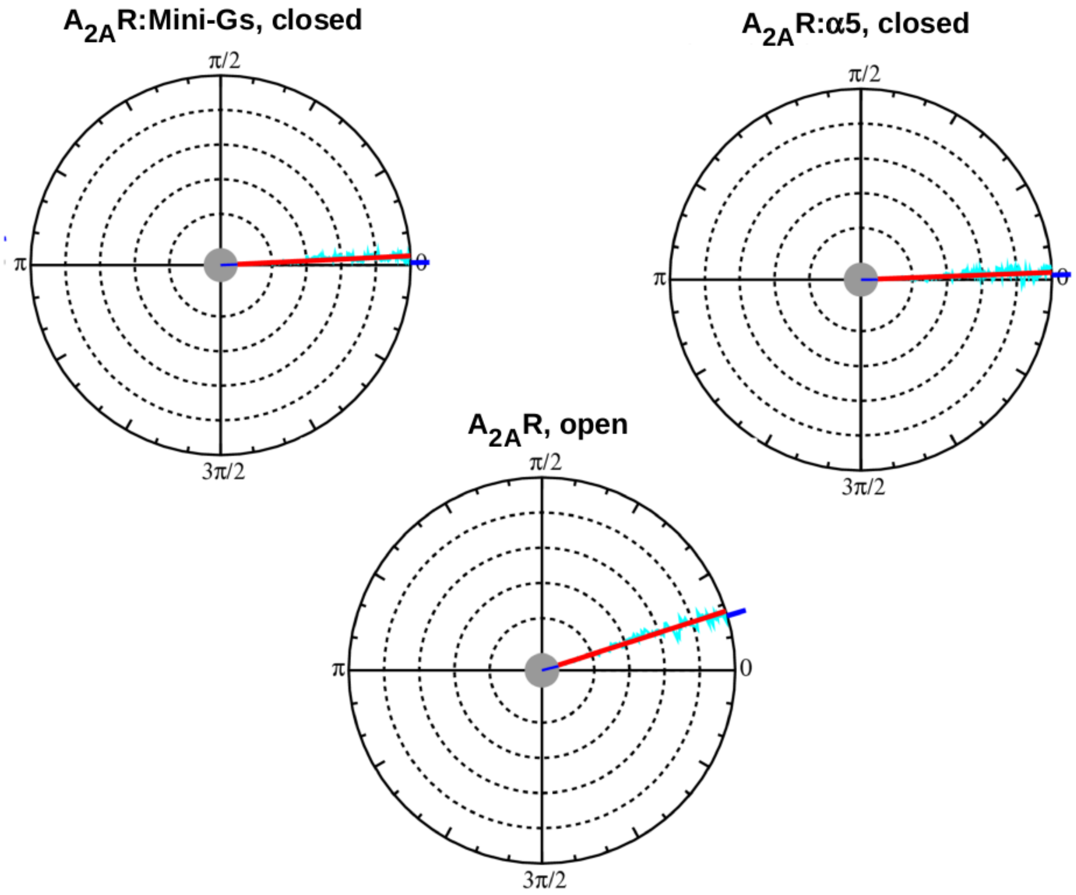

Figure S8. Difference distance matrices for the receptor, based on distances between Cα's. The average distance matrix calculated in A2AR:Mini-Gs:NECA was subtracted from the average distance matrices obtained for A) A2AR, B) A2AR:Mini-Gs and C) A2AR:α5, highlighting conformational changes that led to an open state of the receptor. Distances that increased relative to the A2AR:Mini-Gs:NECA system are indicated in red; decreased distances are represented in blue. In A2AR, the motion of TM2 (red stripe in the region of residues 60-70) contributed to open the orthosteric site. In A2AR:Mini-Gs and A2AR:α5, the lighter colors of the maps indicate pairwise distances similar to those observed in the A2AR:Mini-Gs:NECA system, confirming the allosteric role of Mini-Gs and the α5 helix in maintaining a closed conformation of the agonist-free receptor.

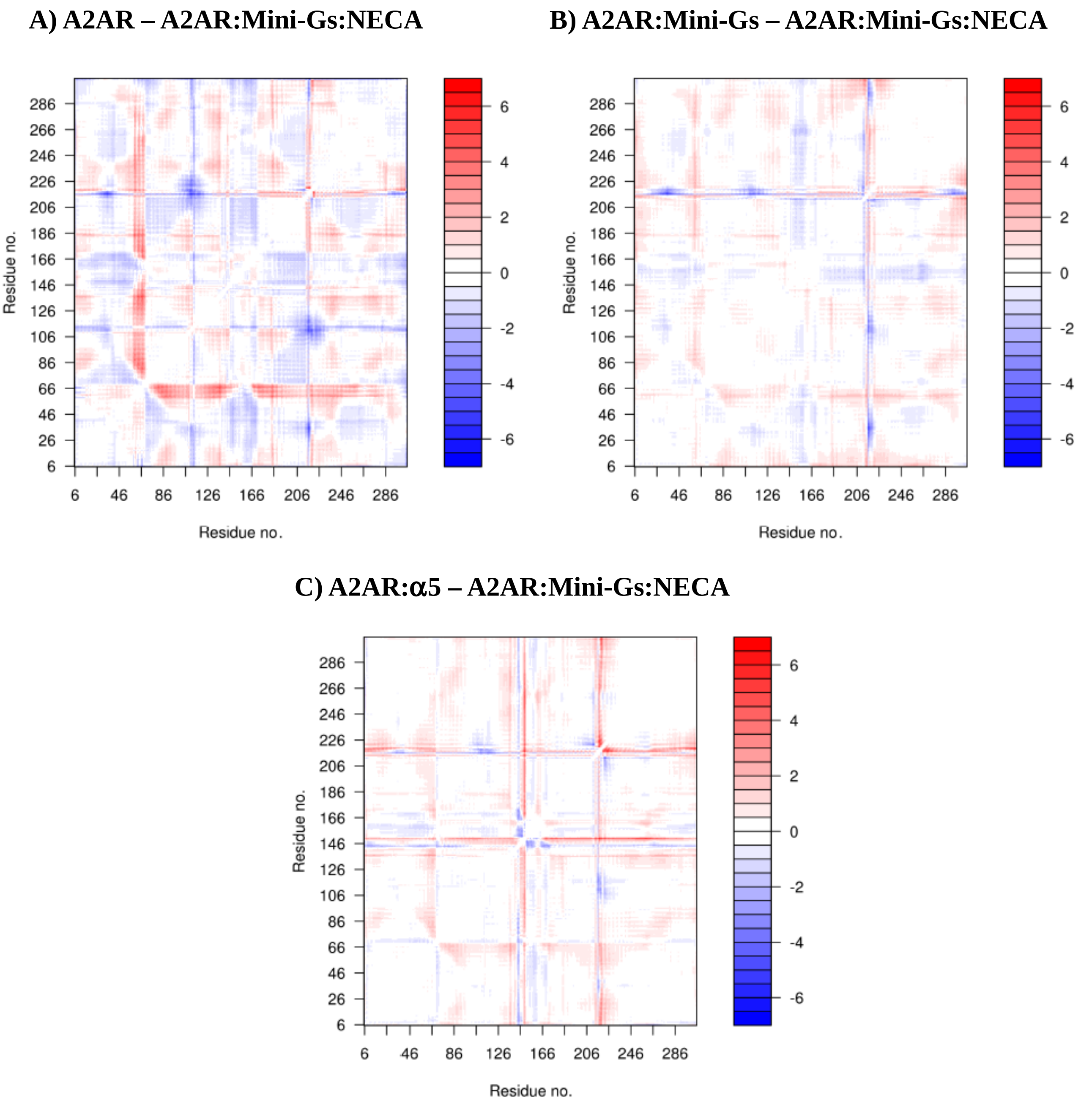

Figure S9. Relaxation of A2AR during the simulations. A) Crystal structure of an inactive form of A2AR (pink; PDB id: 3REY) and conformation of the free A2AR (blue), extracted from the simulations, that corresponds to the minimum RMSD with respect to the crystal structure (3.1 Å, measured on the backbone atoms). An incomplete relaxation of the trajectories towards the inactive X-Ray structure is notably revealed by the conformation of TM6. B) Relaxation of the side chain of ARG 102 upon removal of Mini-Gs. The crystal structure of an inactive form of A2AR is shown in pink (PDB id: 3REY) and a conformation of the free A2AR, extracted from the simulations (that corresponds to the minimum RMSD with respect to the crystal structure) is shown in blue. A representative conformation of the A2AR:Mini-Gs complex (green) is shown for comparison. ARG 102 (shown in licorice representation) adopted a conformation closer to the inactive form in the trajectories of the free receptor. The distal part of TM6 was omitted for clarity.

A

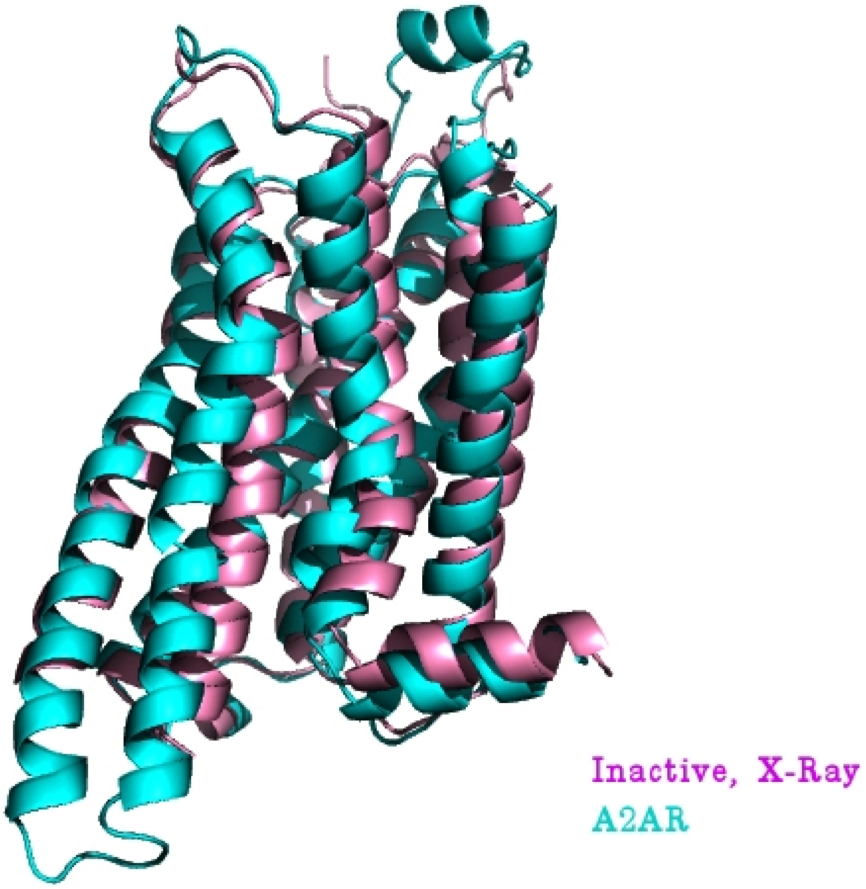

B

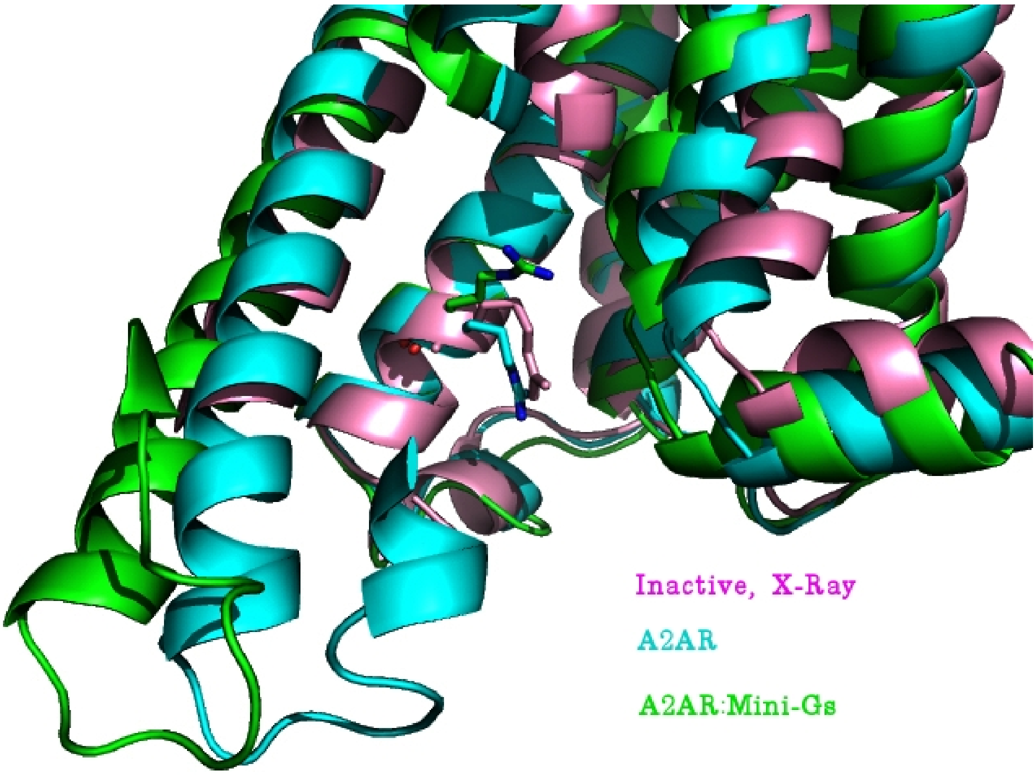

Figure S10. Differences in root mean square fluctuations (RMSF) of the receptor between the form coupled to Mini-Gs and the free form. Regions that are more rigid in the A2AR:Mini-Gs complex are colored in blue; those that present increased flexibility in this complex are shown in red. Note that portions of the extracellular loop 2 (ECL2) are rigidified upon coupling to Mini-Gs.

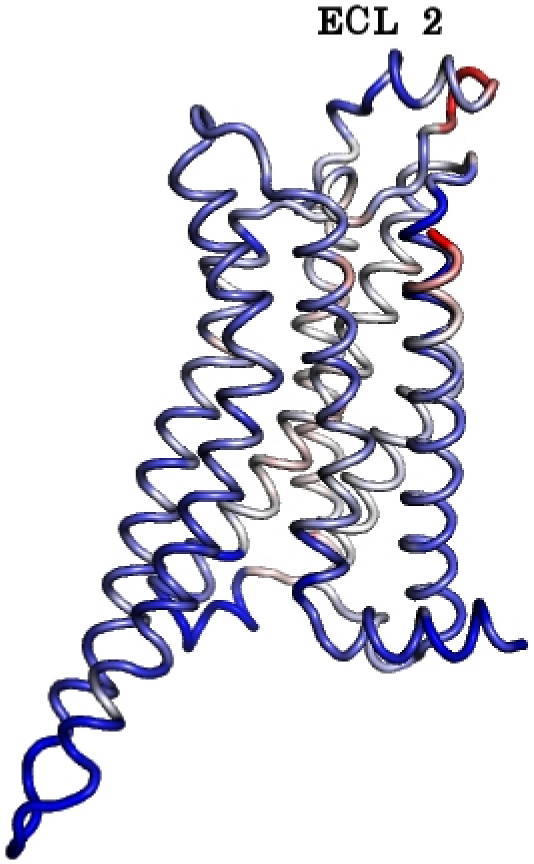

Figure S11. Distribution of the volume of the agonist binding site in distinct simulated conditions, including the double mutant of A2AR (I238A/Y288A) coupled to the α5 helix (purple). The other curves are those shown in figure 2B of the main text. The capacity of the α5 helix to stabilize a more closed conformation of the agonist site (black) was lost in the mutant, where the contact between residues I238 and Y288 was disrupted.

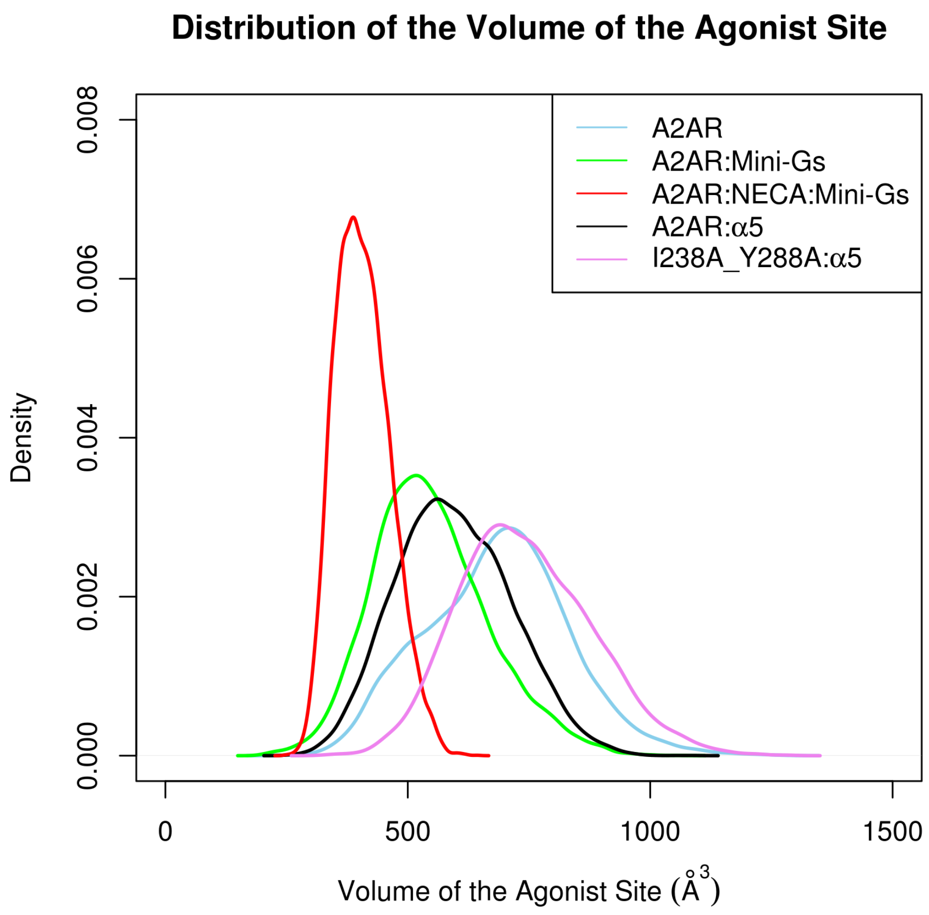

Figure S12. Conformational dynamics of the double mutant of A2AR (I238A/Y288A) coupled to the α5 helix. A) 2D density plot showing the distribution of distances between the Cα's of ALA 63 (2.61) and VAL 84 (3.32) relative to the perimeter of the triangle formed by the Cα of THR 88 (3.36) and the side chains of TRP 246 (6.48) and HIS 278 (7.43). The receptor explored open and intermediate states, but the closed state was abolished (compare with figure 3D). B) Difference distance matrix for the receptor, based on distances between Cα's. The average distance matrix calculated in A2AR_I238A_Y288A:α5 was subtracted from the average distance matrix obtained for A2AR:α5. In the mutant, the motion of TM2 contributed to open the orthosteric site.

A

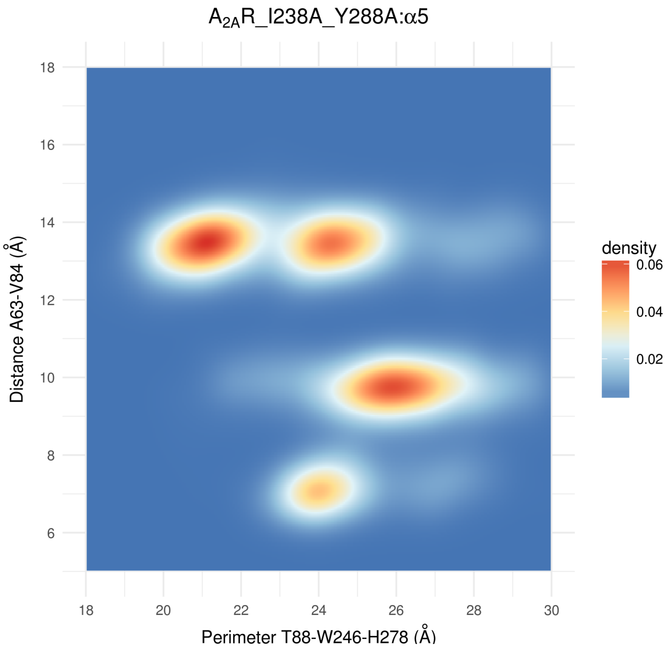

B

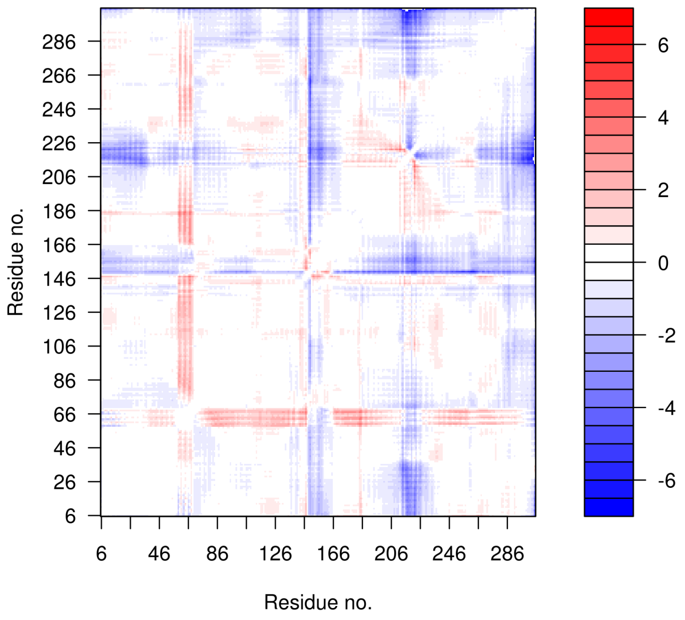

REFERENCES

1. Mezei, M. & Filizola, M. TRAJELIX: A computational tool for the geometric characterization of protein helices during molecular dynamics simulations. *J. Comput. Aided. Mol. Des*. **20,** 97-107 (2006).

2. Mezei, M. Simulaid: a simulation facilitator and analysis program. *J. Comp. Chem.,* **31,** 2658-2668 (2010).
